# Complex effects of testosterone level on ectoparasite load in a ground squirrel: an experimental test for the immunocompetence handicap hypothesis

**DOI:** 10.1186/s13071-024-06261-1

**Published:** 2024-03-30

**Authors:** Li-Qing Wang, Zhi-Tao Liu, Jian-Jun Wang, Yu-Han Fang, Hao Zhu, Ke Shi, Fu-Shun Zhang, Ling-Ying Shuai

**Affiliations:** 1 Grassland Research Institute, Chinese Academy of Agricultural Sciences, Hohhot, China; 2 College of Life Sciences, Harbin Normal University, Harbin, China; 3 Inner Mongolia Autonomous Region Comprehensive Center for Disease Control and Prevention, Hohhot, China; 4 College of Life Sciences, Huaibei Normal University, Huaibei, China

**Keywords:** Immunocompetence handicap, Parasite, Rodent, Sex, Testosterone, Tick

## Abstract

**Background:**

The immunocompetence handicap hypothesis suggests that males with a higher testosterone level should be better at developing male secondary traits, but at a cost of suppressed immune performance. As a result, we should expect that males with an increased testosterone level also possess a higher parasite load. However, previous empirical studies aimed to test this prediction have generated mixed results. Meanwhile, the effect of testosterone level on parasite load in female hosts remains poorly known.

**Methods:**

In this study, we tested this prediction by manipulating testosterone level in Daurian ground squirrels (*Spermophilus dauricus*), a medium-sized rodent widely distributed in northeast Asia. *S. dauricus* is an important host of ticks and fleas and often viewed as a considerable reservoir of plague. Live-trapped *S. dauricus* were injected with either tea oil (control group) or testosterone (treatment group) and then released. A total of 10 days later, the rodents were recaptured and checked for ectoparasites. Fecal samples were also collected to measure testosterone level of each individual.

**Results:**

We found that testosterone manipulation and sex of hosts interacted to affect tick load. At the end of the experiment, male squirrels subjected to testosterone implantation had an averagely higher tick load than males from the control group. However, this pattern was not found in females. Moreover, testosterone manipulation did not significantly affect flea load in *S. dauricus*.

**Conclusions:**

Our results only lent limited support for the immunocompetence handicap hypothesis, suggesting that the role of testosterone on regulating parasite load is relatively complex, and may largely depend on parasite type and gender of hosts.

**Graphical Abstract:**

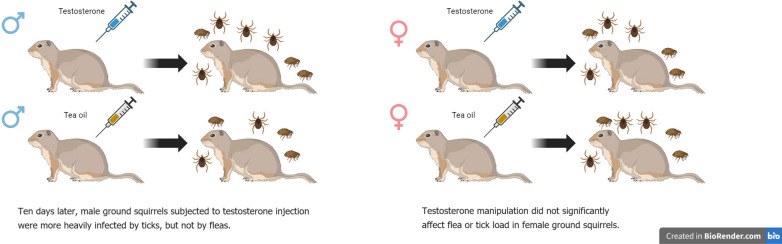

**Supplementary Information:**

The online version contains supplementary material available at 10.1186/s13071-024-06261-1.

## Background

As one of the most fundamental biological processes, parasitism is widespread in terms of both geography and taxonomy [1]. Although in most cases nonlethal to their hosts, parasites often play important roles in shaping behavior, fitness and population dynamics of hosts [2], and may even have keystone effects on community structure in some ecosystems [3]. Meanwhile, many parasites are important transmitters of severe zoonoses including plague and Lyme borreliosis. However, parasites are generally distributed in a nonrandom way. Exploring the distribution patterns of parasites and the mechanisms behind the patterns should contribute to a comprehensive understanding of ecosystem functioning, as well as disease control, human well-being, animal husbandry, and wildlife management [4–7].

A nearly universal pattern in parasitology is that parasites usually show an aggregated distribution [8]. This means that host individuals are often significantly different in their encounter rate or susceptibility to parasites, resulting in a highly variable parasite load among host individuals. Several biological factors of hosts have been found associated with the parasite load, such as body size [9, 10], personality [11], and sex [12]. For example, sex-biased parasitism has been frequently recorded and males are often more heavily infested than females [13, 14]. Several mechanisms have been proposed to explain this pattern. For example, males are usually larger and have a larger home area than females, making them more likely to encounter more parasites or become the preferred targets of parasites. However, male-biased parasitism is not universal and seems to partially depend on parasite types examined [15, 16].

Another interesting mechanism associated with the male-biased parasitism is the immunocompetence handicap hypothesis (ICHH), which states that a higher level of testosterone can promote the production of male secondary traits, but at the cost of decreased immune function [17, 18]. Meanwhile, there is a negative-feedback loop between parasite load and the signal intensity of male secondary trait, as parasitism may hinder the development of several male secondary traits of hosts [19]. As a result, the enhanced expression of male secondary trait may act as an honest signal of “good genes,” i.e., an individual’s ability to withstand a higher parasite burden [17].

A fundamental prediction of the ICHH is that individuals with a higher level of testosterone should also possess a higher parasite load. This prediction has been experimentally tested in many species since the ICHH was proposed. However, the results are largely inconsistent [20], as positive [21–23], negative [24, 25], and non-significant [26–28] association between testosterone level and parasite load have all been repeatedly recorded. The relationship between testosterone level and parasitism seems to be complex and may differ significantly among both host [20] and parasite species [24, 29]. Compared with correlational studies, manipulative experiments should be more effective in detecting the actual role of testosterone level on parasite load.

Moreover, partly because the ICHH was developed to explain the expression of male secondary traits interacted with the immune function and endocrine system, no experimental study has explored the role of testosterone level in shaping the parasite load in females. However, testosterone is not exclusively limited to males, and females may also face a physiological tradeoff associated with testosterone [30, 31]. The association between testosterone level and parasite load in females is poorly known and deserves more investigations [30].

In this study, we explored the effects of testosterone level on the flea and tick load in Daurian ground squirrel (*Spermophilus dauricus*) by manipulating the testosterone level of both male and females. *S. dauricus* is a medium-sized, diurnal rodent widely distributed in grasslands of northeastern Asia [32]. As a well-known host of fleas and ticks, *S. dauricus* often acts as an important transmitter of plague [13, 33]. In a previous study, we found that fleas and ticks possessed distinct distribution patterns on *S. dauricus*, suggesting that the underlying mechanisms of parasitism may also differ between these two types of ectoparasites [13]. For this study, we aimed to address two main questions: (1) would the effects of testosterone level on parasite load differ between fleas and ticks? And, (2) would the effects of testosterone level on parasite load also differ between male and female ground squirrels?

## Methods

### Study area

We carried out our field work in the grassland located within the Experiment Demonstration Base, Grassland Research Institute, Chinese Academy of Agricultural Sciences (40° 36ʹ N, 111° 45ʹ E). This area has a continental temperate monsoon climate, with an average annual rainfall of *ca.* 400 mm and an annual mean temperature of *ca.* 6.9 °C. The dominant plant species are *Leymus chinensis*, *Stipa capillata*, *Cleistogenes squarrosa* and *Medicago sativa*. Based on our own trapping record, *S. dauricus* has been the dominant rodent species here in recent years. Other rodents, such as striped hamster (*Cricetulus barabensis*) and Mongolian gerbil (*Meriones Unguiculatus*), were also recorded but relatively low in abundance. According to our observation, steppe polecats (*Mustela eversmanii*), red foxes (*Vulpes vulpes*) and domestic dogs (*Canis lupus familiaris*) are the major predators feeding on *S. dauricus* [32]. Cattle grazing is common here in spring and summer, resulting in an average grass height of ca. 20 cm and an average vegetation cover of 45%.

### Experimental procedure

In mid-July, 2023, we conducted the first round of live-trapping in two 1-ha sites located in the study area. In this season, most *S. dauricus* were reproductively inactive. The two sites were comparable in terms of vegetation, topography and rodent density. To ensure independence in sampling among the sites, there was a distance of 400 m between the nearest sites. We placed 100 Sherman live traps (arranged in a 10 × 10 grid, with 10-m intervals between neighboring traps) baited with fresh peanuts in each site. Since *S. dauricus* were diurnal, the traps were set open between 07:00 and 19:00 (Beijing time). This round of live-trapping lasted for four consecutive days. We checked all the traps every 2 h and rebaited the traps if needed. All the *S. dauricus* captured were immediately put in separate cotton bags and taken back to our laboratory.

A total of 59 *S. dauricus* (27 males and 32 females) were captured during the first round of live-trapping. We anesthetized each individual by a multi-channel anesthesia machine designed for small animals (R550IE, RWD Life Science Co., Ltd., Shenzhen, China) with isoflurane. To collect the ectoparasites, the body surface of each *S. dauricus* was carefully scanned using a fine-toothed comb and a tweezer. We also checked the inner side of each cotton bag used to contain the ground squirrels. All the ectoparasites collected from a *S. dauricus* were immediately placed in ethanol (95%) contained within a separate 5-ml centrifuge tube. All the *S. dauricus* were weighted to the nearest 0.1 g using an electronic balance, toe-clipped for individual identification, and then maintained in separate plastic boxes for 72 h with access to ad libitum food (peanuts, alfalfa leaves, and commercial pellets) and water. No *S. dauricus* showed any abnormal behavior or healthy problem during this period.

We used all the 52 adult individuals (defined as those heavier than 100 g, 23 males and 29 females) for our formal experiment. Each ground squirrel was randomly assigned to one of two groups: control group (without testosterone injection, 11 males and 14 females) and treatment group (with testosterone injection, 12 males and 15 females). At 15:00–16:00 in the next day after capture, we collected a fresh fecal sample (typically 0.2–0.3 g) from each experimental animal. All the fecal samples were placed in separate 5-ml centrifuge tubes and then immediately stored frozen at −80 °C. About 72 h after capture, each individual was injected intramuscularly with either a dose of tea oil (control group) or a dose of testosterone–oil mixture (10 mg of testosterone undecanoate per ml of tea oil). A total of 1 h after injection, we released all the individuals at the places where they were captured.

A total of 10 days later, we conducted the second round (five consecutive days) of live trapping to recapture the experimental animals. The procedures of live-trapping, anesthesia, ectoparasite collection, fecal sample collection, and animal maintenance were similar to the first round. A total of 28 *S. dauricus* were recaptured (six males and seven females from the control group, and five males and ten females from the treatment group). An experienced taxonomist (Jian-Jun Wang) later identified all the ectoparasites based on dichotomous keys. The whole experimental procedure adhered to the guidelines approved by the American Society of Mammalogists [34] and the Regulations of the Animal Welfare Committee of Beijing Veterinarians of the Agriculture Ministry of China (Beijing, China).

### Hormone extraction and analyses

We typically followed the protocol used by Li et al. [35] to extract testosterone from the fecal samples, with some modifications. A total of 56 fecal samples were used for hormone analyses (i.e., samples collected from the 28 individuals with recaptures, two samples per individual). Since we used wet feces, variations in water content among samples must be accounted for. Therefore, we simultaneously weighed two fecal subsamples (each *ca.* 0.1 g in weight, hereafter subsamples A and B) from each fecal sample. Subsample B was used for measuring water content and was weighed before and after 24-h drying in a drying oven. The water content value was then used to translate the wet sample weight of the relevant subsample A into dry weight.

Subsamples A were used for hormone extraction and placed in separate 10-ml centrifuge tubes. For each tube, we added 4 ml of methanol and 1 ml of distilled water and then vortexed it for 30 min. We then added 2.5 ml of petroleum ether to each tube to remove lipid from it. After 10 min of vortex, each tube was centrifuged at 1500 r/min for 15 min. A total of 2 ml of liquid was drawn from the methanol layer within each tube and then placed into a 5-ml cryopreservation tube. The methanol was dried off under forced air and the remain was used for hormone assay.

We performed testosterone assays with a commercially available enzyme immunoassay kit (Rat Testosterone Elisa Kit, produced by FanYin Biotechnology Co., Ltd., Shanghai, China). This kit has a sensitivity of 1.0 nanomol/l, and < 1% cross-reactivity to other steroids (including progestins, corticoids and estrogens). The testosterone levels were reported as nanogram of fecal testosterone per gram of dry feces.

### Statistical analyses

We performed all the statistical work in R platform 4.2.2 [36]. We first adopted a paired-sample *t*-test to test whether our experimental treatment affected the fecal testosterone level of *S. dauricus*. We built a negative-binomial generalized linear mixed-effect model (GLMM) on tick load recorded on the recaptured individuals (hereafter TickLoad_after_) using the R package “lme4” [37] and “lmerTest” [38]. The fixed terms included treatment (control or treatment group), sex (male or female), body weight (averaged value of the two measurements), tick load recorded in the first round of ectoparasite check (i.e., tick load before the testosterone manipulation, hereafter TickLoad_before_), flea load in the second round of ectoparasite check (hereafter FleaLoad_after_), and an interactive term between treatment and sex. Site ID was used as a random term. Similar models were also built for FleaLoad_after_, with fixed factors including treatment, sex, body weight, flea load recorded in the first round of ectoparasite check (hereafter FleaLoad_before_), TickLoad_after_, and an interaction between treatment and sex. Variance inflation factors (VIFs) were calculated using the R package ‘car’ [39] to assess multicollinearity. As the VIFs were all smaller than ten (Table 1), we retained all the factors in the models. Model selection was performed based on Alkaike Information Criterion corrected for small sample size (AIC_c_) [40] using the R package “MuMIn” [41]. Since the performance did not differ significantly between top candidate models (i.e., delta AIC_c_ smaller than 2), we used conditional model averaging to get an “averaged model” based on the full set of candidate models [40]. As we detected a significant interactive effect between treatment and sex on TickLoad_after_, we also built two GLMMs on TickLoad_after_ for male and female squirrels separately (Table 2). For these two models, the fixed terms included treatment, body weight, TickLoad_before_, and FleaLoad_after_.

**Table 1:** Results of conditional model averaging, based on negative binomial generalized linear mixed-effects model on ectoparasite load (whole data set) of Daurian ground squirrels (*Spermophilus dauricus*)

Model/factors	Estimate	Standard error	*Z* value	*P* value	*VIF*
TickLoad_after_					
Intercept	23.15	20.88	1.07	0.28	NA
FleaLoad_after_	2.16	1.17	1.74	0.082	1.32
Sex (male)	7.31	20.22	0.34	0.73	2.40
Treatment (testosterone)	− 2.62	18.63	0.13	0.89	2.48
**Sex: treatment**	61.13	27.58	2.10	0.036	2.84
TickLoad_before_	0.39	0.29	1.29	0.20	1.44
Body weight	−0.23	0.17	1.31	0.19	1.26
FleaLoad_after_					
Intercept	3.32	3.38	0.94	0.35	NA
TickLoad_after_	0.050	0.027	1.73	0.084	3.20
Sex (male)	1.87	2.70	0.66	0.51	2.69
Treatment (testosterone)	1.78	2.47	0.68	0.50	1.87
Sex: treatment	0.56	4.38	0.12	0.90	5.50
FleaLoad_before_	0.41	0.27	1.41	0.15	3.10
Body weight	0.018	0.033	0.50	0.62	2.90

TickLoad_after_ and FleaLoad_after_ refer to tick load and flea load recorded in the second round of ectoparasite check, respectively. TickLoad_before_ and FleaLoad_before_ refer to tick load and flea load recorded in the first round of ectoparasite check, respectively. Variance inflation factor (VIF) for each explanatory factory is indicated. Significant terms marked by bold

**Table 2:** Results of conditional model averaging, based on negative binomial generalized linear mixed-effects model on tick load of male or female Daurian ground squirrels (*Spermophilus dauricus*)

Model/factors	Estimate	Standard error	*Z* value	*P* value	*VIF*
Male					
Intercept	3.69	0.37	8.69	< 0.001	NA
**Treatment (testosterone)**	0.98	0.33	2.43	0.016	1.12
FleaLoad_after_	0.041	0.024	1.41	0.16	1.39
TickLoad_before_	0.0024	0.010	0.19	0.85	1.10
Body weight	− 0.0027	0.0070	0.31	0.76	1.33
Female					
Intercept	3.35	0.57	5.62	< 0.001	NA
Treatment (testosterone)	0.18	0.39	0.43	0.67	1.69
FleaLoad_after_	0.013	0.049	0.24	0.81	1.39
TickLoad_before_	0.0083	0.0053	1.42	0.16	1.51
Body weight	−0.0054	0.0035	1.38	0.17	1.29

TickLoad_after_: tick load recorded in the first round of ectoparasite check. FleaLoad_after_: flea load recorded in the second round of ectoparasite check. Variance inflation factor (VIF) for each explanatory factory is indicated. Significant terms marked by bold

## Results

On the 28 squirrels used for analyses, we collected a total of 534 ticks and 73 fleas before testosterone manipulation, and 1108 ticks and 132 fleas in the second round of ectoparasite check (Additional file 1). Before testosterone manipulation, no significant difference in tick load was detected between males and females (Wilcoxon test: *W* = 79, *P* = 0.50). After testosterone manipulation, males were more heavily infested by ticks than females (Wilcoxon test: *W* = 47.5, *P* = 0.033). The diversity of ectoparasites was rather low, as only one tick species (*Haemaphysalis verticalis*) and one flea species (*Citellophilus tesquorum mongolicus*) were recorded. The prevalence of fleas was 57.14% (16/28) and 64.29% (18/28) in the first and the second round of ectoparasite check, respectively. The prevalence of ticks was 67.86% (19/28) and 100% in the first and the second round of ectoparasite check, respectively.

**Fig. 1:**
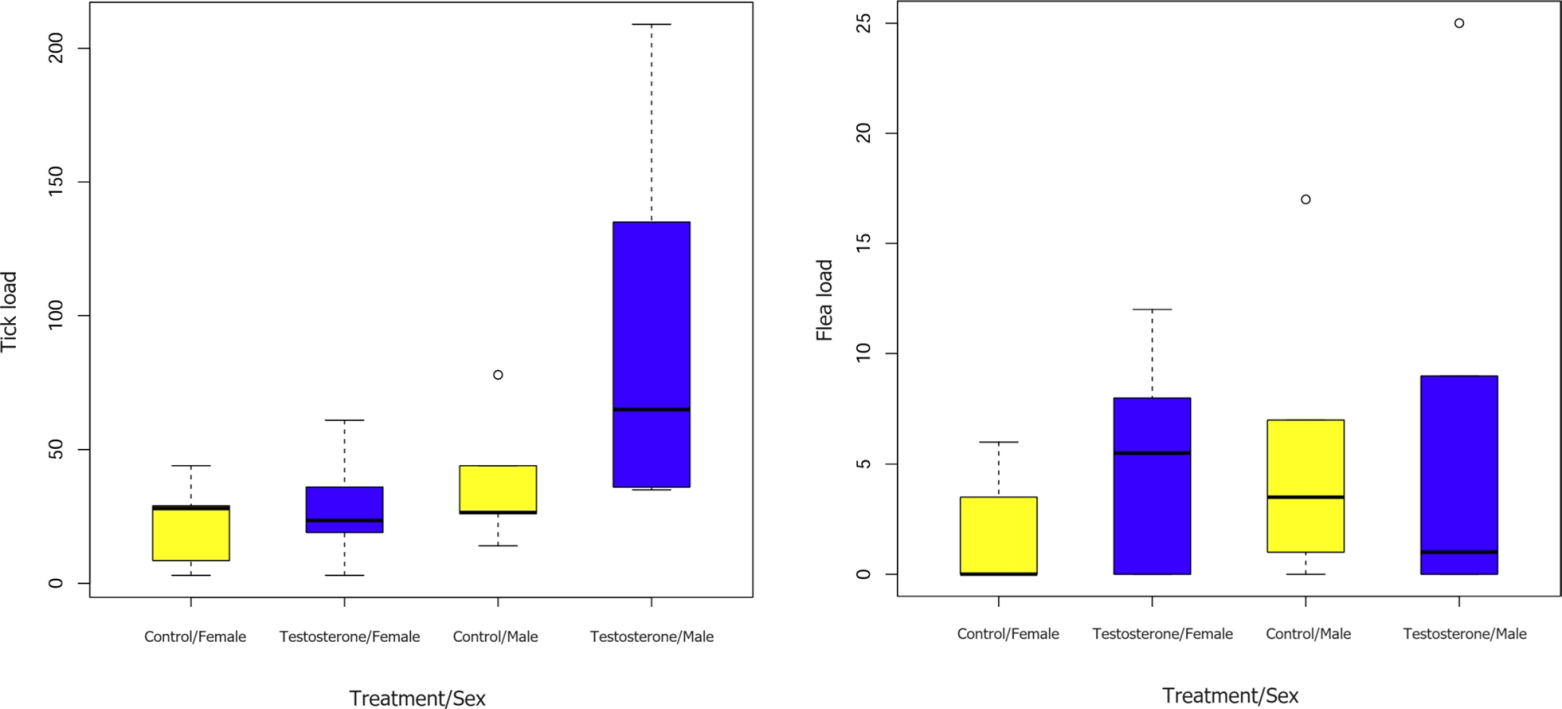
Boxplots indicating the interactive effects of sex (male/female) and treatment (testosterone/control) on tick load and flea load in Daurian ground squirrels (*Spermophilus dauricus*)

Compared to the testosterone level prior to the experimental manipulation, the squirrels subjected to testosterone injection showed a significantly increased testosterone level after the manipulation (Wilcoxon test: *W* = 198, *P* < 0.001), while those subjected to tea oil injection did not show such a change in testosterone level (Wilcoxon test: *W* = 98, *P* = 0.50). According to the averaged GLMM, sex and treatment significantly interacted to affect TickLoad_after_ (Table 1, Fig. 1). We also detected a marginally positive association between flea load and tick load after testosterone manipulation (Table 1). However, no significant association was found between the explanatory factors and FleaLoad_after_. For male squirrels, individuals subjected to testosterone injection possessed a significantly higher TickLoad_after_ (Table 2). However, such a trend did not exist in female squirrels (Table 2).

## Discussion

In consistence with the ICHH, we found that male squirrels subjected to testosterone implantation later possessed a significantly higher tick load than those from the control group. Several mechanisms may contribute to this pattern. First, increased androgens often make animals more active and more aggressive [42], thus increasing their chance of encountering other individuals, as well as their exposure to parasites transmitted by conspecifics. Second, as the ICHH states, while increased testosterone level helps to develop male secondary traits and bring some reproductive benefits, it may also cause increased host susceptibility to parasites through immunosuppression [43]. Of course, these mechanisms are not mutually exclusive. To further disentangle the roles of behavioral changes and immunosuppression in shaping parasite abundance, it is important to simultaneously monitor behavioral pattern and immune functions of hosts, which is the aim of our next-step work.

As documented before, male-biased parasitism is not a universal pattern. This is also the case for the association between testosterone level and parasite infestation. In this study, testosterone treatment did not seem to significantly affect flea load in *S. dauricus*. In our previous work, we detected no sex difference in flea load in this rodent [13]. Similarly, Kowalski et al. [44] also found that flea load did not differ significantly between male and female yellow-necked field mice (*Apodemus flavicollis*). Difference in behavior and life history between ticks and fleas may partly explain their difference in parasitism. Compared with ticks, fleas are generally more mobile, more sensitive to environmental changes and need to lay eggs in the host burrows. As many other mammals do, female adult *S. dauricus* solitarily nurse their offsprings. Meanwhile, unlike males, female *S. dauricus* usually built their nests with relatively soft and fine bedding materials, which may help them to maintain the ambient temperature within the nests [45]. As a result, the burrow of a female adult *S. dauricus* with its cubs should be more attractive for fleas than a male’s burrow [46]. Moreover, living with cubs may also promote transmission of fleas between mother and cubs [47]. These mechanisms may thus counterbalance the male-biased pattern or the positive effect of testosterone level on parasitism.

Although the androgen-mediated trade-off between reproductive success and health has been repeatedly reported in males, it was much less studied in females. In a previous work on female meerkats, Smyth et al. [30] found a positive association between fecal testosterone level and the abundance of nematodes. This is not the case in our study, as we detected no significant effect of testosterone manipulation on ectoparasite load in female squirrels. Two reasons may contribute to this difference: first, unlike female meerkats, female *S. dauricus* are solitary and may not rely on increased androgen level to maintain its dominance status or stay in a central position within its social network. As a result, the increased testosterone level should be less effective to facilitate the female squirrels’ exposure to parasites. Second, the study on female meerkats is by essence a natural experiment and based on correlational analyses, which is unable to reveal the exact causal relationship between testosterone level and parasite load, especially when changes in parasite load also trigger changes in testosterone level.

## Conclusions

The ICHH has long been used to explain the widespread sexual differences in parasite load. However, this hypothesis remains controversial to some extent [20], partly because one of its fundamental prediction—the positive relationship between testosterone level and parasite load- is not consistently supported by empirical studies. In this study, we tested this prediction by manipulating testosterone level in a medium-sized rodent. We found that testosterone supplementation had a positive effect on tick load in males, but not in females. Moreover, testosterone manipulation did not significantly affect flea load in *S. dauricus*. In summary, our results suggested that the role of testosterone on regulating parasite load is relatively complex, and may largely depend on parasite type and gender of hosts. The lack of generality in the testosterone effect is reasonable to some extent, as testosterone can shape parasite load in multiple ways, such as changing encounter rate of parasites through behavioral alteration, and modulating resistance to parasites through many physiological processes (e.g., impairing antibody production, and interacting with corticosteroids), which are not always immunosuppressive. These mechanisms can take effect together, and the overall effect of testosterone should depend on biological traits of both hosts and parasites. It is thus important to simultaneously track testosterone-related changes in behavioral mode (e.g., social behavior, home range, and activity level) and physiological status (e.g., number of antibodies, number of white blood cells, and glucocorticoids level) of both male and female hosts, if we intend to disentangle the roles of various mechanisms in regulating parasite load. An up-to-date meta-analysis or global synthesis is also required to grasp the global trend of testosterone effect, and to explain the heterogeneity existing among the various studies.

### Supplementary Information

**Additional file 1: Table S1.** Original data used for analyses in this study.

## Data Availability

The dataset used for analyses in this study can be found in the supplementary information.
